# Tumor of the Iris with Cataract—Its Removal by Operation

**Published:** 1872-12-15

**Authors:** Chas. L. Stoddard

**Affiliations:** Whitewater, Wisconsin


### TUMOR OF THE IRIS WITH CATARACT — ITS REMOVAL BY OPERATION.

BY CHAS. L. STODDARD, M. D., WHITEWATER, WISCONSIN.

Mrs. Brownley, aged 45, presented herself at my office, about two years ago, complaining of pain in her right eye. I found at the lower part of the iris a slight discoloration, but could not determine at the time that it indicated disease, as it did not differ in appearance from maculæ often found on the iris. Six months later I again saw her, and found a cataract, well developed, and total blindness of that eye as the result. The iris, at this time, differed only in appearance by the maculæ being somewhat more conical in appearance, and encroaching on the anterior chamber. She still complained of pain in the eye, for which I prescribed, but did not advise operative interference. Again, in the winter of this year, she consulted me, when I found a well-marked tumor, adherent to the lower anterior part of the iris, of a blackish or brownish appearance, pressing forward the iris, into contact with the posterior cornea, almost, and pressing the opaque lens backward, giving the pupil a peculiar appearance, as of a hollow tube. I now advised her to have the tumor removed, and that a consultation might be held on her case with some surgeon of eminence, as I could not decide whether the case was likely to assume a malignant form or not. An oculist of eminence, from Milwaukee, advised, on consultation, that the whole eyeball be removed, but she would not consent; neither did I advise so severe an operation, but was inclined rather, to remove the tumor and lens by extraction, and an iridectomy of all that part of the iris involved in the mass. To this plan she finally consented. After considering the difficulties to be encountered, owing to the obliteration of the anterior chamber, and the adhesion which the tumor might have formed with the anterior structures, I decided on a modification of the linear incision; I entered my knife in the sclerotic, near the corneal junction, passed it through the iris, without touching the pupil (I did not dilate it), and between the tumor, as nearly as possible, and lens, divided the ciliary ligament on the inner or nasal side, and then passed it outwards, through the sclerotic, making, in fact, a combination of the old and new or linear incision, together with a division of the ciliary ligament and iris. I then carefully closed the lids, with a compress, for a few seconds. On opening and making slight pressure, a body escaped, covered on one side with blackish pigment, and on the other had a fleshy or raw surface. It was as large as a pea, of the consistence of soft cheese, and was, in fact, the veritable tumor, complete. Again, on making slight pressure, the lens escaped, being compressed at one part, where it lay in contact with the tumor, and appeared to be merely the very much thickened capsule. There was a slight escape of vitreous matter, but not sufficient to interfere with a good result. She was treated in the usual manner after an ordinary extraction. Two days later incision healed, and anterior chamber filled up pretty well. Eight days after operation, all going on well — eye well-filled, pupil, normal in shape; cornea perfectly clear; a slight coagulate still remaining undissolved in the anterior chamber, below the pupillary margin, but could distinguish objects quite clearly, so that the operation may be considered a success, both as to the removal of the tumor and restoration of sight, as she is quite healthy otherwise. The tumor on examination appeared to be a fleshy mass, coming under the head of fibrous or fatty—in fact, appeared more like a glandular structure than any other. The microscope showed nothing to indicate that it was of a malignant character—simple cells only, imbedded in ordinary strome, and fewer of a fatty kind than I had anticipated from its physical characteristics.

The facts in this case will fully corroborate the theories of modern oculists, who show that the anterior eye may be freely divided at almost any part; that the iris may be incised, or removed in part; the ciliary ligament freely divided; and, in fact, any modification or incision made in appropriate cases with hopes of a good result.

